# Prevalence and Perceived Preventability of Self-Reported Adverse Drug Events – A Population-Based Survey of 7099 Adults

**DOI:** 10.1371/journal.pone.0073166

**Published:** 2013-09-04

**Authors:** Katja Marja Hakkarainen, Karolina Andersson Sundell, Max Petzold, Staffan Hägg

**Affiliations:** 1 Nordic School of Public Health NHV, Gothenburg, Sweden; 2 Department of Public Health and Community Medicine, University of Gothenburg, Gothenburg, Sweden; 3 Centre for Applied Biostatistics, Sahlgrenska Academy, University of Gothenburg, Gothenburg, Sweden; 4 Division of Clinical Pharmacology, Linköping University, Linköping, Sweden; University of Maryland, School of Medicine, United States of America

## Abstract

**Purpose:**

Adverse drug events (ADEs) are common and often preventable among inpatients, but self-reported ADEs have not been investigated in a representative sample of the general public. The objectives of this study were to estimate the 1-month prevalence of self-reported ADEs among the adult general public, and the perceived preventability of 2 ADE categories: adverse drug reactions (ADRs) and sub-therapeutic effects (STEs).

**Methods:**

In this cross-sectional study, a postal survey was sent in October 2010 to a random sample of 13 931 Swedish residents aged ≥18 years. Self-reported ADEs experienced during the past month included ADRs, STEs, drug dependence, drug intoxications and morbidity due to drug-related untreated indication. ADEs could be associated with prescription, non-prescription or herbal drugs. The respondents estimated whether ADRs and STEs could have been prevented. ADE prevalences in age groups (18–44, 45–64, or ≥65 years) were compared.

**Results:**

Of 7099 respondents (response rate 51.0%), ADEs were reported by 19.4% (95% confidence interval, 18.5–20.3%), and the prevalence did not differ by age group (p>0.05). The prevalences of self-reported ADRs, STEs, and morbidities due to drug-related untreated indications were 7.8% (7.2–8.4%), 7.6% (7.0–8.2%) and 8.1% (7.5–8.7%), respectively. The prevalence of self-reported drug dependence was 2.2% (1.9–2.6%), and drug intoxications 0.2% (0.1–0.3%). The respondents considered 19.2% (14.8–23.6%) of ADRs and STEs preventable. Although reported drugs varied between ADE categories, most ADEs were attributable to commonly dispensed drugs. Drugs reported for all and preventable events were similar.

**Conclusions:**

One-fifth of the adult general public across age groups reported ADEs during the past month, indicating a need for prevention strategies beyond hospitalised patients. For this, the underlying causes of ADEs should increasingly be investigated. The high burden of ADEs and preventable ADEs from widely used drugs across care settings supports redesigning a safer healthcare system to adequately tackle the problem.

## Introduction

Improving patient safety and reducing preventable patient harm, including adverse drug events (ADEs), is emphasised by national and global health authorities [1], [2]. An ADE is commonly defined as “*an injury resulting from medical intervention related to a drug*” [3], although other definitions exist [4], [5]. In hospitals, 4–5% of patients experience ADEs [4], [6], including ADE-related hospitalisations and ADEs occurring during hospitalisation. In previous studies, 11–86% of ADEs among outpatients being hospitalised [7]–[9], and 15–90% of ADEs among inpatients [6], [9]–[11] are estimated preventable. However, current evidence on ADEs is largely limited to inpatients and voluntary reports of health professionals. Although patients outside hospitals report ADEs not detected otherwise [12], few and often small studies have investigated patient-reported ADEs [13]–[22], and no studies have investigated ADEs in a representative sample of the general population. Further, few studies have defined sub-categories of ADEs, other than adverse drug reactions (ADRs), even though other types of ADEs have been identified [23]–[31]. Thus, we conducted a population-based survey study to estimate the 1-month prevalence of self-reported ADEs, sub-categories of ADEs, and two sub-categories of preventable ADEs (ADRs and sub-therapeutic effects of drug therapy (STEs)) among the adult general public in Sweden. Other aims were to assess the perceived preventability of ADRs and STEs by the general public, and to identify drug classes and organ systems associated with self-reported ADEs.

## Methods

### Ethics Statement

An ethical approval for the study was received from the Regional Ethics Board in Gothenburg (archive number 238-10). Participants provided a written consent to participate through responding to a postal survey, accompanied with an introductory letter that was in accordance with the Declaration of Helsinki.

### Study Design and Sample

**Figure 1:**
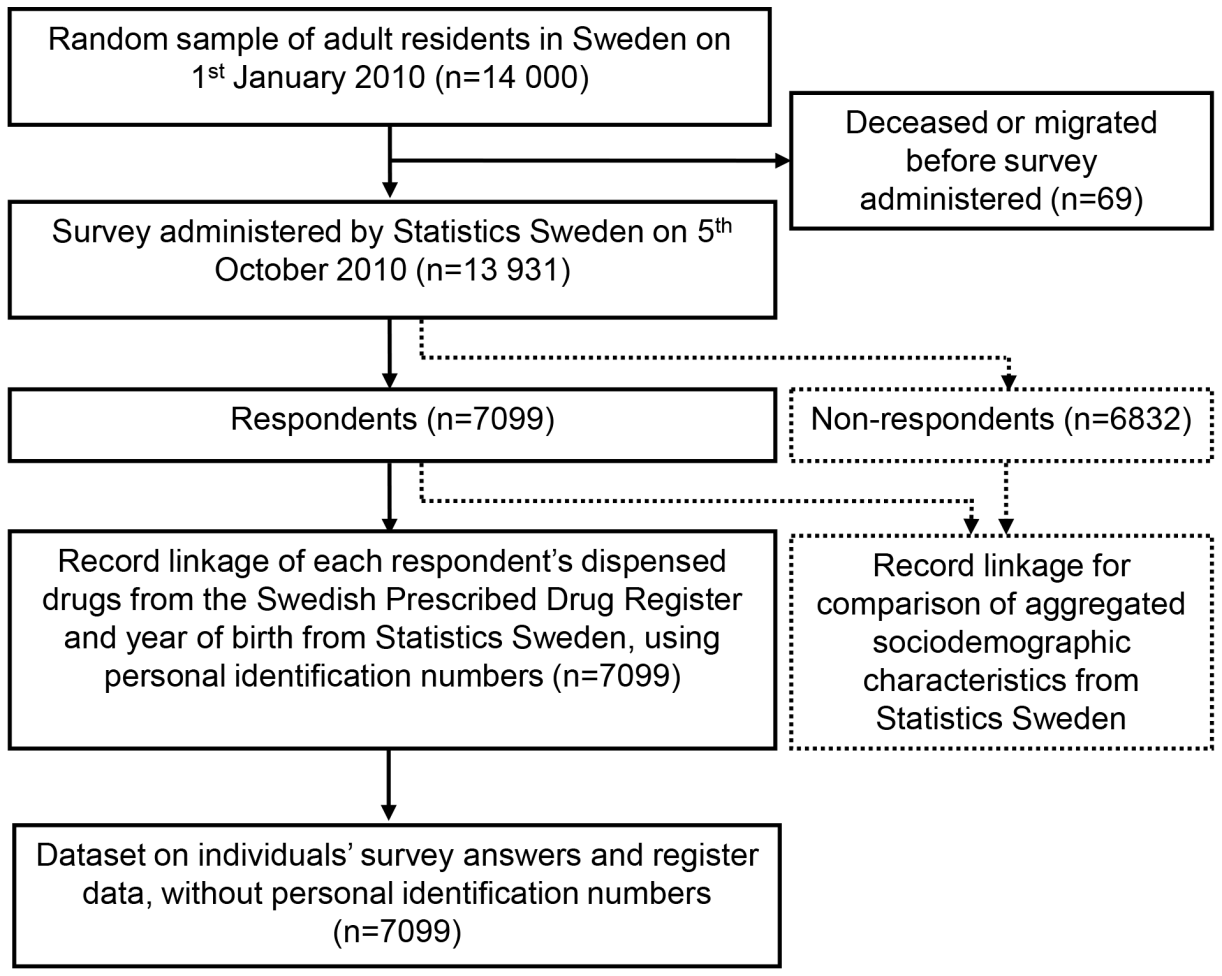
Study flow diagram.

This cross-sectional study among the adult general public in Sweden combines survey and register data (Fig. 1). The survey was mailed to random sample of 13 931 residents aged ≥18 years, drawn from the Total Population Register at Statistics Sweden. The sample size was based on an estimated 10% 1-month prevalence of self-reported ADRs, slightly higher than in a previous Swedish survey reporting a 2-week prevalence of 6.4% [13], and an expected preventability of 10% [15], [19]. Requiring a maximum length of a 95% confidence interval of +/−0.3% unit, and expecting a 60% response rate [13], the calculated minimum number of respondents was n = 7 043. The sample size was doubled to n = 14 000 individuals, allowing more detailed analyses.

### Definitions

An ADE was defined as “*an injury resulting from medical intervention related to a drug”* [3], and ADEs could be associated with prescription, non-prescription or herbal drugs. Preventability was defined according Hallas as “*the drug event was due to a drug treatment procedure inconsistent with presentday knowledge of good medical practice or was clearly unrealistic, taking the known circumstances into account*“ (definitely preventable) or “*the prescription was not erroneous, but the drug event could have been avoided by an effort exceeding the obligatory demands*” (possibly preventable) [32]. Some have previously considered ADEs to consist of non-preventable ADRs, and medication errors that are by definition preventable [33], while others have defined ADR differently and considered part of them preventable [34], [35]. In the current study, ADRs were defined according to the World Health Organization [36], [37] for designing the survey question, were included in ADEs, and could also be preventable. Although “*injury*” in the broad definition for ADEs [3] could be interpreted to include a range of other events other than ADRs, and medication errors such as omission of a dose [38] may result in other events, other event categories are not detailed in most studies on ADEs. Instead, previous studies on self-reported ADEs have used a general question on all ADEs, such as: “*After taking your prescription medication(s), have you had any problems or symptoms?*” [19]. As a general question on ADEs was considered to have been ambiguous for laymen to interpret and the current study aimed at identifying sub-categories of ADEs, our research group identified other ADE categories from the literature on drug-related injuries [23]–[31]. In the event categorisation, we aimed at classifying types of morbidity or injury, rather than reasons for it. The identified ADE categories, in addition to ADRs, were: drug intoxications from overdose [28], drug dependence [27], STEs [29]–[31], and morbidity due to drug-related untreated indication [23]–[26]. Prior to designing the survey, these ADE categories were defined partially based on the literature [32], [39], [40], and partially based on the decision of the research group (Table S1). Modifying a previous definition [32], we defined STEs as an absence of therapeutic response that could be linked causally either to (prescribed) dose that was too low, to drug non-compliance, recent dose reduction/discontinuation or inadequate monitoring, or to improper drug selection. Further, STEs could also occur when the treatment has been rational (e.g. first line treatment or best available medicines were not effective enough). Definitions for the other ADE categories are described in Table S1. Each of the ADE categories could be preventable but were not automatically considered preventable.

### Survey Design

We examined previous studies of self-reported ADEs in the survey development [13], [15], but none of the previous questionnaires was found directly applicable for the aims of our study. Thus, the research group developed new questions for laymen on experienced ADEs during the past month, based on the pre-defined definitions (Table S1). The respondents were requested to describe symptoms of and/or drugs associated with ADEs, depending on the ADE category as detailed in Table S1. As a result of pilot-tests for face and content validity in different populations, including health professionals, immigrants, and the elderly, the questions were simplified from the original definitions, as it became evident that laymen do not read long instructions and definitions. Simplification was also considered necessary for minimising the risk of non-response and reporting biases among persons with language, health literary or cognitive barriers. For the same reason, the length of the survey was minimised, which was why preventability was asked only for the two ADE categories that were expected to be the most frequent: ADRs and STEs. Based on the definition for preventability [32], and after several rounds of piloting, the final question on the perceived preventability of each self-reported ADR read (in Swedish): “*Do you think you or someone else could have done something to prevent this side effect? For example at the time of prescribing the drug, purchase of the drug, use of the drug, or monitoring of the treatment. If yes, describe what.*” The corresponding question for each STE was: “*Do you think you or someone else could have done something for reaching a sufficient effect from the drug? For example at the time of prescribing the drug, purchase of the drug, use of the drug, or monitoring of the treatment. If yes, describe what.*” Other response alternatives were “*no*” and “*don’t know*”.

The survey included questions on whether the respondents had healthcare encounters during the past month, as described in detail elsewhere [41]. In short, the encounters could be with nurse or physician, visits or telephone contacts, outpatient or inpatient, and specialised or not, and excluded dental care. We also asked whether the respondents had during the past month used prescription, non-prescription or herbal drugs, each including pre-defined drug categories for laymen to comprehend and an additional free text field for other drugs.

The questionnaire, an introduction letter and a prepaid return envelope were mailed by Statistics Sweden in October 2010. One postal card reminder (October 2010) and two reminders including the questionnaire (November 2010, January 2011) were sent.

### Coding

Handwritten self-reported symptoms of and drugs associated with ADEs were transcribed and coded (KMH), according to a prespecified coding protocol. All reported ADEs were included in the analyses, including when the respondent could not specify the event (n = 31), unless if it was evident from the answer that the question was misunderstood (Table S1). ADRs and STEs were considered preventable when respondents had answered “*yes*” on preventability and/or described perceived actions for preventing the ADR or STE. Because the causality and preventability assessments were done by the respondents, we use the terms “self-reported ADEs” and “perceived preventability”.

However, some cases required interpretation, for example when several ADEs were reported in the same free text field or the same ADE in multiple fields. Interpretation was also required for coding unspecific self-reported symptoms, such as “feeling lazy”, and when answers on the nominal question on preventability (“yes”/“no”/“I don’t know”) and the free text field on preventability contradicted. When such interpretations were required, a senior clinical pharmacologist (SH) verified the coding. To further improve the coding, an additional researcher independently coded 10% of all responses. After comparing the double-coding with the first coding (KMH), the coding protocol was clarified and all cases re-coded accordingly (KMH).

### Register Data

Survey and register data were linked using the personal identity number (Figure 1). The respondents’ year of birth was retrieved from the Total Population Register. Variables from the national Swedish Prescribed Drug Register [42] on drugs dispensed to the respondents included the date of dispensing and the drug code according to the Anatomical Therapeutic Chemical (ATC) Classification System [43]. The drug register covers all drugs dispensed in Swedish pharmacies but excludes drugs bought over-the-counter, or administrated in hospitals or other facilities with own drug distribution.

Aggregated data on the respondents’ and non-respondents’ (linkage using the personal identity number), and the sampling frame’s socio-demographics were retrieved from Statistics Sweden: age in three groups; marital status as married or registered partnership, single, divorced or widowed; area of residence as cities including commuting municipalities or others; country of birth as born in Sweden or not, and born in an Organisation for Economic Co-operation and Development (OECD) country or not; highest level of education in mandatory school (6–9 years), secondary/high school (2–3 years after mandatory school) or high education (≥1 years after secondary school); and yearly individual income in quintiles (defined in 2009).

### Analyses

The aggregated socio-demographic variables from Statistics Sweden were reported descriptively for the respondents, non-respondents and sampling frame (Table 1). Differences between the characteristics of the respondents and non-respondents were tested statistically, using t-test for mean ages and χ^2^ test for categorical variables.

**Table 1:** Characteristics of the study population (n = 13 931) compared to the adult general population in Sweden, retrieved from Statistics Sweden.

Variable	Study population			Adult general populationn (%)
	Respondents n (%)	Non-respondents n (%)	P-value^a^	
**Total**	7099 (51.0)	6832 (49.0)		7 382 226
**Age** b				
mean (SD)	53.2 (18.2)	45.4 (18.9)	<0.001^c^	49.3 (18.9)
18–44 years	2432 (34.3)	3674 (53.8)	<0.001^c^	3 196 795 (43.3)
45–64 years	2508 (35.3)	2011 (29.4)	<0.001^c^	2 415 481 (32.7)
≥65 years	2159 (30.4)	1147 (16.8)	<0.001^c^	1 769 906 (24.0)
Missing	0 (0)	0 (0)		44 (0.0)
**Sex**				
Female	3839 (54.1)	3117 (45.6)	<0.001^c^	3 737 939 (50.6)
Missing	0 (0)	0 (0)		44 (0.0)
**Marital status** b				
Single	2231 (31.4)	3226 (47.2)	<0.001^c^	2 841 758 (38.5)
Married or registered partnership	3505 (49.4)	2424 (35.5)	<0.001^c^	3 179 760 (43.1)
Divorced	869 (12.2)	802 (11.7)	0.36^d^	885 669 (12.0)
Widowed	494 (7.0)	308 (5.6)	0.001^c^	474 995 (6.4)
Missing	0 (0)	0 (0)		44 (0.0)
**Area of residence** b				
Cities and commuting municipalities	5260 (73.7)	5164 (75.6)	0.04^c^	5 440 849 (73.7)
Missing	0 (0)	0 (0)		5368 (0.1)
**Country of birth**				
Other than Sweden	819 (11.5)	1504 (22.0)	<0.001^c^	1 226 097 (16.6)
Non-OECD country	403 (5.7)	929 (13.6)	<0.001^c^	669 341 (9.1)
Missing	0 (0)	0 (0)		955 (0.0)
**Highest level of education** e				
Mandatory school	1499 (21.1)	1804 (26.4)	<0.001^c^	1 680 087 (22.8)
Secondary/high school	3054 (43.0)	3091 (45.2)	<0.001^c^	3 267 860 (44.3)
High education	2499 (35.2)	1734 (25.4)	<0.001^c^	2 254 400 (30.5)
Missing	47 (0.7)	203 (3.0)		179 879 (2.4)
**Yearly individual income** f				
0–11769 EUR	1368 (19.3)	2248 (32.9)	<0.001^c^	1 994 505 (27.0)
11770–19112 EUR	1461 (20.6)	1267 (18.5)	0.002^c^	1 446 496 (19.6)
19113–25703 EUR	1482 (20.9)	1279 (18.7)	0.001^c^	1 448 504 (19.6)
25704–33706 EUR	1494 (21.0)	1109 (16.2)	<0.001^c^	1 334 734 (18.1)
33707 EUR –	1294 (18.2)	929 (13.6)	<0.001^c^	1 157 987 (15.7)
Missing	0 (0)	0 (0)		0 (0)

OECD = Organisation for Economic Co-operation and Development; EUR = Euro.

a For statistical significance between respondents and non-respondents using t-test for comparing mean ages and χ^2^ test for comparing categorical variables.

b September 24^th^ 2010, just before the survey was sent.

c Significant difference between the respondents and non-respondents.

d Non-significant difference between the respondents and non-respondents.

e In 2010.

f In 2009 before taxation. Yearly average exchange rate in 2009 from Swedish krona to Euro 10.6213.

The 1-month prevalences of different ADE categories, with 95% confidence intervals (CI), were calculated for all respondents in the main analysis. Individuals with at least one ADE of each category were used in the numerator and the total number of respondents in the denominator. All respondents were chosen as the denominator, because ADEs could occur without dispensed drugs (non-prescription drugs and drugs from previous stockpile), morbidities due to drug-related untreated indications and STEs did not require drug use, and prolonged symptoms of ADEs can be experienced without current drug use. As sensitivity analyses, the prevalences were also calculated using two alternative denominators: respondents with self-reported prescription, non-prescription or herbal drug use during the past month; and respondents who reported at least one healthcare encounter during the past month. In further sensitivity analyses, we investigated how the total ADE prevalence was influenced by omitting morbidities due to drug-related untreated indications and non-preventable STEs from ADEs. This was done using all respondents, respondents with drug use and respondents with healthcare encounters as denominators. For preventability, the number of preventable ADRs and STEs were divided by the number of all ADRs and STEs.

The prevalence of each ADE category, using all respondents in the denominator, was calculated in three age groups and compared using χ^2^ or Fisher’s exact test: aged 18–44, 45–64, and 65 years or more. Age 45 was chosen as a cut-off, because cardiovascular mortality [44] and drug use [45], among other health conditions, increase in Sweden at this age. After the retirement age of 65, morbidities further increase and the proportion of the population with dispensed drugs exceeds 80% [42]. To investigate the robustness of the age categorisation, the prevalences were also compared in age groups with 5-year intervals. STATA software version 10 was used in all analyses.

When calculating drugs associated with ADEs, the total number of ADEs of the category was the denominator. Drugs were categorised according to the ATC Classification System [43], including all main groups (1^st^ level) and pharmacological subgroups (3^rd^ level) representing >1% of the ADE category, and chemical substances (5^th^ level) representing ≥20% of the given pharmacological subgroups. Psycholeptics (antipsychotics, anxiolytics, hypnotics and sedatives) (N05) and psychoanaleptics (antidepressants, psychostimulants, agents used for ADHD, nootropics, psycholectics and psychoanaleptics in combination, and anti-dementia drugs) (N06) were also categorised into the 4^th^ level drug classes. ADE categories associated with herbal drugs without an ATC code were reported. For comparing drugs associated with ADEs to common drugs, the most common drugs dispensed to all respondents during six months before returning the survey were described, using data from the Swedish Prescribed Drug Register. For each respondent, all drugs with a unique ATC code [43] dispensed between six months before the survey return date and the return date were included. Dispensed drugs during six months was considered to appropriately reflect on drugs that could have caused ADEs during the study period, because regularly used drugs are prescribed for three months in Sweden and doubling the three months allowed non-adherence. A 6-month period enabled also capturing prescriptions for irregular use, such as analgesics, that were filled before and used during the 1-month study period. Because of the dominance of antibiotics in yearly statistics on dispensed drugs, a full calendar year was considered unsuitable for reflecting drug classes potentially causing ADEs during the 1-month study period.

For ADRs, the System Organ Classes and Preferred Terms, i.e. individual symptoms, were determined according to the Medical Dictionary for Regulatory Activities (MedDRA) [46], and presented when they represented ≥1% of all or preventable ADRs. Chemical substances [43] associated with the reported Preferred Terms at least twice were reported.

## Results

### Study Population

In total, 7099 responded giving a response rate of 51.0% (Table 1). The non-respondents were younger, more commonly men and born outside Sweden, lived more commonly alone and in urban areas, and had lower level of education and income.

### Prevalence

In the main analyses, 19.4% (95% CI, 18.5–20.3%) of all respondents reported at least 1 ADE during the past month (Table 2). The prevalence did not differ in the three age groups for all ADEs, ADRs, drug intoxications, and morbidities due to drug-related untreated indications. For drug dependence and STEs, the prevalence differed in the three age groups (p<0.001 respective p = 0.001), also when investigated in age groups with 5-year intervals. Of the 1377 respondents reporting ADEs, 54.2% reported 1, 35.9% 2–3, and 9.9% 4–11 ADEs.

**Table 2:** 1-month prevalence of self-reported ADEs and preventable ADEs, by ADE category and age group (n = 7099).

	Aged 18–44 years (n = 2432)		Aged 45–64 years (n = 2508)		Aged ≥65 years (n = 2159)			All ages (n = 7099)	
	n	Prevalence % (95% CI)	n	Prevalence % (95% CI)	n	Prevalence % (95% CI)	P-value^b^	n	Prevalence % (95% CI)
**Any ADE** a	**482**	**19.8 (18.2–21.4)**	**487**	**19.4 (17.8–21.0)**	**408**	**18.9 (17.2–20.5)**	**0.73** c	**1377**	**19.4 (18.5–20.3)**
Adverse drug reactions	184	7.6 (6.5–8.6)	200	8.0 (6.9–9.0)	170	7.9 (6.7–9.0)	0.86^c^	554	7.8 (7.2–8.4)
Drug intoxications from overdose	3	0.1 (0.0–0.3)	7	0.3 (0.1–0.5)	4	0.2 (0.0–0.4)	0.49^c^	14	0.2 (0.1–0.3)
Drug dependences	23	1.0 (0.6–1.3)	56	2.2 (1.7–2.8)	79	3.7 (2.9–4.5)	<0.001^d^	158	2.2 (1.9–2.6)
Sub-therapeutic effects of drug therapy	219	9.0 (7.9–10.1)	188	7.5 (6.5–8.5)	132	6.1 (5.1–7.1)	0.001^e^	539	7.6 (7.0–8.2)
Morbidities due to drug-relateduntreated indications	207	8.5 (7.4–9.6)	210	8.4 (7.3–9.5)	158	7.3 (6.2–8.4)	0.28^c^	575	8.1 (7.5–8.7)
**Preventable adverse drug reactions or sub-** **therapeutic effects of drug therapy** a	**75**	**3.1 (2.4–3.8)**	**83**	**3.3 (2.6–4.0)**	**50**	**2.3 (1.7–3.0)**	**0.11** c	**208**	**2.9 (2.5–3.3)**
Preventable adverse drug reactions	28	1.2 (0.7–1.6)	35	1.4 (0.9–1.9)	29	1.3 (0.9–1.8)	0.73^c^	92	1.3 (1.0–1.6)
Preventable sub-therapeutic effects ofdrug therapy	53	2.2 (1.6–2.8)	54	2.2 (1.6–2.7)	24	1.1 (0.7–1.6)	0.010^f^	131	1.8 (1.5–2.2)

ADE = adverse drug event; CI = confidence interval.

a As one person could have multiple ADEs, the combined prevalence is lower than the sum of the prevalences of the ADE categories.

b For testing the statistical significance between all three age groups using χ^2^ test, with the exception of using Fisher’s exact test for drug intoxications from overdose due to low number of cases.

c Non-significant difference between all the three age groups.

d Significant difference between all three age groups; for all pairwise age group comparisons p<0.01.

e Significant difference between all three age groups; for a pairwise comparison 18–44 vs. ≥65 years p<0.001; other pairwise age group comparisons p>0.05.

f Significant difference between all three age groups; for pairwise comparisons 18–44 vs. ≥65 years and 45–64 vs. ≥65 years p<0.01; for 18–44 vs. 45–64 years p>0.05.

One or more preventable ADRs or STEs were reported by 208 respondents (Table 2), resulting in a combined prevalence of 2.9% (2.5–3.3%). The prevalence did not differ by age group for all preventable ADEs and preventable ADRs (p = 0.11 respective p = 0.73), but did for preventable STEs (p = 0.010). Of the 208 respondents with preventable ADRs or STEs, 70.7% reported 1, 25.5% 2–3, and 3.8% 4–6 preventable ADEs.

In the sensitivity analyses using respondents with drug use as the denominator (Table 3), the prevalence of all ADEs and its subcategories increased slightly compared to the main analysis. Of all 1377 respondents reporting ADEs, 189 (13.7%) reported healthcare encounters. Among the respondents with healthcare encounters, the prevalences of ADRs and STEs increased from the main analysis, while the prevalences of the other ADE categories remained similar to the main analyses. When morbidities due to drug-related untreated indications were omitted from ADEs, the prevalences of ADEs were 14.7% (13.9–15.6%) among all respondents, 17.6% (16.6–18.6%) among respondents with drug use, and 29.6% (25.9–33.3%) among respondents with healthcare encounters. The prevalence decreased further when both morbidities due to drug-related untreated indications and non-preventable STEs were omitted from ADEs, being 10.7% (10.0–11.4%) for all respondents, 12.7% (11.9–13.6%) for respondents with drug use, and 22.3% (18.9–25.7%) for respondents with healthcare encounters.

**Table 3:** Sensitivity analyses for the 1-month prevalence of self-reported ADEs and preventable ADEs, varying the denominator.

	Main analysis:		Sensitivity analyses:			
	Denominator all respondents(n = 7099)		Denominator respondentswith self-reported drug use^b^(n = 5798)		Denominator respondents withself-reported healthcareencounters (n = 578)	
	n	Prevalence % (95% CI)	n	Prevalence % (95% CI)	n	Prevalence % (95% CI)
**Any ADE** a	**1377**	**19.4 (18.5–20.3)**	**1318**	**22.7 (21.7–23.8)**	**189**	**32.7 (28.9–36.5)**
Adverse drug reactions	554	7.8 (7.2–8.4)	540	9.3 (8.6–10.1)	106	18.3 (15.2–21.5)
Drug intoxications from overdose	14	0.2 (0.1–0.3)	14	0.2 (0.1–0.4)	1	0.2 (–0.2–0.5)
Drug dependences	158	2.2 (1.9–2.6)	154	2.7 (2.2–3.1)	20	3.5 (2.0–5.0)
Sub-therapeutic effects of drug therapy	539	7.6 (7.0–8.2)	528	9.1 (8.4–9.8)	89	15.4 (12.4–18.3)
Morbidities due to drug-relateduntreated indications	575	8.1 (7.5–8.7)	535	9.2 (8.5–10.0)	55	9.5 (7.1–11.9)
**Preventable adverse drug reactions or sub-** **therapeutic effects of drug therapy** a	**208**	**2.9 (2.5–3.3)**	**204**	**3.5 (3.0–4.0)**	**38**	**6.6 (4.5–8.6)**
Preventable adverse drug reactions	92	1.3 (1.0–1.6)	91	1.6 (1.2–1.9)	14	2.4 (1.2–3.7)
Preventable sub-therapeutic effectsof drug therapy	131	1.8 (1.5–2.2)	128	2.2 (1.8–2.6)	27	4.7 (2.9–6.4)

ADE = adverse drug event; CI = confidence interval.

a As one person could have multiple ADEs, the combined prevalence is lower than the sum of the prevalences of the ADE categories.

b Self-reported use of prescription, non-prescription or herbal drugs during the 1-month study period for which the respondents reported ADEs.

### Categories of ADEs and Perceived Preventability

Of all reported 2578 ADEs, 847 (32.9%) were ADRs, 20 (0.8%) drug intoxications, 174 (6.7%) drug dependences, 745 (28.9%) STEs, and 792 (30.7%) morbidities due to drug-related untreated indications. Reported 139 preventable ADRs and 167 preventable STEs resulted in preventability estimates of 16.4% (13.9–18.9%) and 22.4% (19.4–25.4%), respectively. The combined preventability for both was 19.2% (14.8–23.6%).

### Associated Drugs

Nervous system drugs were the most commonly associated with ADRs (33.2%), drug dependences (93.7%), drug intoxications (50.0%), and STEs (32.0%) (Table 4). Within nervous system drugs (Table S2), antidepressants were the most common among ADRs (15.4%), analgesics among STEs (16.6%), hypnotics and sedatives among drug dependences (53.7%), and analgesics among intoxications (26.7%).

**Table 4:** Drug classes^a^ associated to self-reported adverse drug events (ADEs), ordered according to the most commonly dispensed drugs to all respondents.

Drug class^a^	Dispensed to allrespondents^b^ (n = 7099),n (%)	ADRs (n = 847),n (%)	Sub-therapeuticeffects of drug therapy(n = 745), n (%)	Drug dependences(n = 174), n (%)	Drug intoxicationsfrom overdose (n = 20),n (%)
Cardiovascular system	2093 (29.5)	163 (19.2)	52 (7.0)	0 (0)	6 (30.0)
Nervous system	1700 (23.9)	281 (33.2)	238 (32.0)	163 (93.7)	10 (50.0)
Alimentary tract and metabolism	1501 (21.1)	34 (4.0)	70 (9.4)	0 (0)	0 (0)
Blood and blood forming organs	1219 (17.2)	24 (2.8)	10 (1.3)	0 (0)	2 (10.0)
Genito urinary system and sex hormones	1109 (15.6)	53 (6.3)	24 (3.2)	0 (0)	0 (0)
Respiratory system	1110 (15.6)	56 (6.6)	73 (9.8)	0 (0)	0 (0)
Antiinfectives for systemic use	1077 (15.2)	42 (5.0)	24 (3.2)	0 (0)	2 (10.0)
Musculo-skeletal system	895 (12.6)	61 (7.2)	111 (14.9)	1 (0.6)	1 (5.0)
Systemic hormonal preparations^c^	667 (9.4)	46 (5.4)	14 (1.9)	0 (0)	0 (0)
Dermatologicals	633 (8.9)	10 (1.2)	42 (5.6)	0 (0)	0 (0)
Sensory organs	554 (7.8)	5 (0.6)	5 (0.7)	0 (0)	0 (0)
Antineoplastic and immunomodulatingagents	178 (2.5)	73 (8.6)	12 (1.6)	0 (0)	0 (0)
No dispensed drugs during thepast 6 months	2417 (34.0)	NA	NA	NA	NA
ATC not available^d^	NA	115 (11.9)	69 (9.3)	10 (5.8)	0 (0)

ADE = adverse drug event; ADR = adverse drug reaction; ATC = Anatomical Therapeutic Chemical; NA = not applicable.

a Categorised according to the Anatomical Therapeutic Chemical (ATC) Classification System [43] main groups (1^st^ level) representing >1% of the ADE category.

b Dispensed drugs from the Swedish Prescribed Drug Register, including all drugs with a unique the Anatomical Therapeutic Chemical (ATC) Classification System [43] code for each respondent, dispensed from six months before the survey return date until the return date.

c Excluding sex hormones and insulins.

d Drug missing or unclear, or complementary medicine.

Preventable ADRs were the most commonly from drugs for the nervous system (30.9%), cardiovascular system (20.9%), and genitourinary system and sex hormones (7.9%). Preventable STEs were most commonly reported from drugs for the nervous system (35.9%), musculoskeletal system (10.8%), and alimentary tract and metabolism (9.6%). As for all ADRs and STEs, antidepressants were the most common subgroup among preventable ADRs (12.2%), and analgesics among preventable STEs (15.0%).

### Organs Affected by ADRs

ADRs were the most frequently gastrointestinal (26.9%) or general disorders (17.5%) (Tables 5 and S3). Affected organ systems and the main drug classes, subgroups and chemical substances for all and preventable ADRs and STEs were similar.

**Table 5:** Organ systems and symptoms affected by all and preventable self-reported adverse drug reactions (ADRs).

Organ systems^a^ and symptoms^b^	ADRs (n = 847) n (%)	Preventable ADRs (n = 139) n (%)
**Gastrointestinal disorders**	**228 (26.9)**	**28 (20.1)**
Nausea	55 (6.5)	9 (6.5)
Diarrhoea	32 (3.8)	2 (1.4)
Dry mouth	30 (3.5)	–
Abdominal pain upper	28 (3.3)	4 (2.9)
Gastric disorder	24 (2.8)	4 (2.9)
Constipation	22 (2.6)	4 (2.9)
Vomiting	–	2 (1.4)
**General disorders and administration site conditions**	**148 (17.5)**	**17 (12.2)**
Fatigue	94 (11.1)	8 (5.8)
Pain	–	4 (2.9)
Hyperhidrosis	–	3 (2.2)
**Nervous system disorders**	**98 (11.9)**	**17 (12.2)**
Dizziness	33 (3.9)	8 (5.8)
Headache	23 (2.7)	3 (2.2)
Tremor	12 (1.4)	–
Hypoaesthesia		2 (1.4)
**Psychiatric disorders**	**97 (11.5)**	**20 (14.4)**
Anxiety	20 (2.4)	3 (2.2)
Insomnia	15 (1.8)	4 (2.9)
Depressed mood	14 (1.7)	5 3.6)
**Skin and subcutaneous tissue disorders**	**43 (5.1)**	**12 (8.6)**
Pruritus	10 (1.3)	3 (2.2)
Alopecia	9 (1.1)	–
Rash	–	3 (2.2)
**Musculoskeletal and connective tissue disorders**	**43 (5.1)**	**10 (7.2)**
Muscle spasms	11 (1.3)	3 (2.2)
**Cardiac disorders**	**32 (3.8)**	**7 (5.0)**
Palpitations	10 (1.2)	–
Dizziness	9 (1.1)	–
**Respiratory, thoracic and mediastinal disorders**	**31 (3.7)**	**5 (3.6)**
Epistaxis	–	2 (1.4)
**Reproductive system and breast disorders**	**29 (3.4)**	**5 (3.6)**
Erectile dysfunction	–	2 (1.4)
Metrorrhagia	–	2 (1.4)
**Vascular disorders**	**17 (2.0)**	**6 (4.3)**
**Investigations**	**14 (1.7)**	**2 (1.4)**
Weight increased	14 (1.7)	2 (1.4)
**Renal and urinary disorders**	**14 (1.6)**	**–**
**Eye disorders**	**10 (1.2)**	**3 (2.2)**
**Metabolism and nutrition disorders**	**9 (1.1)**	**2 (1.4)**
Increased appetite	–	2 (1.4)
**Immune system disorders**	**–**	**2 (1.4)**

ADR = adverse drug reaction.

a Representing ≥1% of all or preventable ADRs. According to the System Organ Classes according to the Medical Dictionary for Regulatory Activities (MedDRA) [46]. For 11 (1.30%) ADRs, the System Organ Class could not be determined.

b Representing ≥1% of all or preventable ADRs. According to the Preferred Terms of the Medical Dictionary for Regulatory Activities (MedDRA) [46].

## Discussion

Our 19% 1-month prevalence of self-reported ADEs among the general public was comparable to previous studies with 18–25% ADE prevalences during 3–12 months, among mainly elderly outpatients [15], [18]–[22]. However, considering our younger study population and shorter study period, our prevalence was relatively high, although our ADE definition and the inclusion of ADEs from non-prescription drugs were expected to increase our prevalence. When we excluded morbidities due to drug-related untreated indications and non-preventable STEs from ADEs, to mimic a previous definition for ADEs [15], [19], the ADE prevalence decreased to 11%, which is still considerable. Although possible over-reporting may have resulted in overestimating our ADE prevalence, our prevalence was probably also decreased by recall bias, underreporting of asymptomatic ADEs, and potentially poor response rate among person with likely high ADE burden, including persons in hospitals [4], [6] and residential care [47], [48], and the frail elderly [49]. Acknowledging the factors that may have increased and decreased our prevalence, our study does indicate that ADEs are a significant burden in the general population, which is also suggested by previous expert panel studies [50], [51].

The lack of previous similar studies hinders comparing our prevalences of different ADE categories to previous findings. Differing study periods may explain our higher ADR prevalence compared to a 2-week prevalence [13], and our lower prevalence compared to an annual prevalence among the elderly [16]. The use of a checklist for ADR symptoms may have caused a higher ADR prevalence of “current” ADRs in one study [17], although a causality assessment decreased markedly the prevalence. The prevalence of self-reported drug dependences in our study was high compared to a lower monthly prevalence of non-therapeutic use of prescription narcotic and addictive drugs (0.6% for men and 1.3% for women) previously estimated in Sweden [52], which may indicate an overestimation in our study, if respondents interpreted the question as being dependent on a drug. Alternatively, the difference may be due to excluding dependence when drug treatment was appropriate in the previous study [52]. Among the few self-reported drug-intoxications in our study, serious cases must be underrepresented, such as fatal intoxications [28], because hospitalised patients are less likely to reply to a home-posted survey. STEs and morbidities due to drug-related untreated indications have previously been common among hospitalised and emergency care patients [26], [29], [30], [53]–[56], and in expert panel studies investigating all patients with medical care [50], [51], [57]. However, our prevelences of STEs and morbidities due to drug-related untreated indications, and their proportions of all ADEs, were relatively high compared to the previous studies [26], [29], [30], [53]–[56]. Despite the possible misclassification of ADEs between the categories and possible over- and under-reporting, our study demonstrates that drug-related injuries of different nature are frequently experienced by the adult general public.

The drug classes associated with the ADE categories differed partially, but the majority of self-reported ADEs were attributable to commonly used drugs, as described previously [15]. Drugs for the nervous system were the most commonly associated with all categories of self-reported ADEs, similarly to previous findings [13], [16], [17], [52], [58]. Within the drug class, pharmacological groups varied by ADE category: ADRs were dominated by antidepressants, STEs by analgesics, and drug dependence by hypnotics and sedatives. Drugs for the cardiovascular system were commonly associated with self-reported ADRs, as found previously [16], [17], [58], but self-reported STEs of cardiovascular drugs were not, although described frequent in emergency care [31]. This is probably due to the respondents’ limited capability to recognise STEs of drugs for non-symptomatic conditions and prophylaxis, leading to their underestimations. Nonetheless, the differing patterns of ADE-related drugs indicate that categorising ADEs provides additional information on their nature, compared to reporting all ADEs together. Thus, categorising ADEs may enhance investigating their preventability and contributing factors.

ADRs reported by the general public in our study expectedly differed from ADRs causing admissions [59], [60], or occurring in hospitals [61]. NSAIDs, diuretics, warfarin and antidiabetics have commonly been associated with ADR-related admissions [59], [62], while we found these drugs less common. Drugs described to commonly cause ADRs occurring during hospitalisation include loop diuretics, opioids, systemic corticosteroids, inhaled beta-agonists, oral anticoagulants, and antibiotics [61], differing from our self-reports. However, antidepressants and in particular selective serotonin reuptake inhibitors appear a preeminent cause of ADRs across settings, as they are a common cause of ADR-related admissions [59], and dominate ADRs in our and others’ studies outside hospitals [15], [63]. In hospitals, ADRs commonly result in hepatic, renal and hematologic disorders, bleedings, hypoglycemia and electrolyte disturbances [59], [61], [62], while we and others [13], [16] found self-reported ADRs more commonly gastrointestinal, general disorders, related to the nervous system, psychiatric, or dermatological. If symptomless, ADRs common among inpatients may be under-reported in our study, or respondents may have reported e.g. hypoglycemia as tremor causing an under-estimation of hypoglycemia. However, our results do suggest that the general public experiences a large quantity of ADRs that are not captured in studies among inpatients.

The general public’s perceived preventability of ADRs and STEs was lower than in most previous studies [6]–[11], [50], [51], perhaps due to the respondents’ limited capability to judge preventability. Although preventable ADEs due to errors related to clinical decision making were probably under-reported, our study provides valuable information on the burden of preventable ADEs due to other, patient-experienced errors, for example in self-administration. However, we found reported drugs and affected organs for all and preventable events similar, as in studies in hospitals [6], [64], [65], indicating that understanding preventable ADEs requires other research methods. Thus, future studies should investigate the underlying causes of preventable ADEs reported by patients using qualitative methods. Studying factors related to the health system and using root cause analyses have, for example, been suggested [66], [67].

The differing prevalences and nature of self-reported ADEs and their sub-categories in our study compared to studies in various care settings indicate that combining self-reports from the public to clinical data would be advantageous for investigating ADEs in the future. As most self-reported ADEs were experienced without healthcare encounters, including a telephone contact, and few ADEs involved a hospitalisation, a large proportion of ADEs are probably overlooked in studies investigating exclusively patients, in particular inpatients. Even though we were unable to assess seriousness, the self-reported ADEs among the general public without healthcare encounters were most likely less serious than ADEs in hospitals. As a large quantity of non-serious adverse events in hospitals have previously been found to consume more resources than fewer serious adverse events [68], non-serious ADEs in the general population may also consume considerable resources, for example in primary care or through decreased productivity [41]. In studies in oncology, patients have reported ADRs, such as fatigue and nausea, earlier and more frequently than clinicians [69], patient-reports have correlated better to the overall health status [69], and clinicians have underestimated the impact of the ADRs on the patients’ daily life [70]. The general public’s perception on preventability is also likely to complement practitioners’ preventability assessments based clinical data, as clinical data probably lack information on patients’ perception on, for example, adequate patient-clinician communication and errors in drug use outside care units. Thus, self-reported ADEs and their perceived preventability should not be ignored from investigations of the prevalence, nature and prevention of ADEs. However, studies relying exclusively on self-reports, like ours, reflect to some extent reporting behaviour and patient satisfaction, and are limited by recall, reporting, and non-response biases and the inability to assess causality and preventability against clinical data,. For improving the understanding of ADEs and their prevention from both clinical and patient perspectives, ADEs and their preventability should be studied using both self-reports and clinical data for the same population sample.

Once ADEs are better understood, interventions for their prevention, detection and mitigation may be developed [71]. Successful interventions for reducing preventable ADEs include computerised prescribing aids [72] and clinical pharmacists’ participation in physician rounds in hospitals [73], to name some. The design, implementation and evaluation of interventions for improving safety and quality must be carefully planned, as introducing new interventions may alter the pattern of errors without necessarily improving safety [74], [75], and the lack of resources probably hinders adopting all possible interventions. Further, as simplifying and standardising complex systems are known to improve safety [71], [76], [77], adding complexity to an already complex healthcare may decrease safety [76]. Instead, improving patient safety significantly has been argued to require large-scale efforts to redesign a safer healthcare system [77]–[79], including implementing generic, cross-sectional and specific interventions. Although there are many solutions to reduce ADEs, the high burden of ADEs and preventable ADEs from widely used drugs across care settings supports, in our view, the argument for system redesign to adequately tackle the problem.

### Methodological Considerations

The varying definitions for ADEs [4], [5] and preventability [80] in the literature limit comparison of studies in the field. Because our study was driven by a research question on the burden of ADEs in the general population, we chose a broader definition for ADEs than in most previous studies. Our ADE categorisation, however, enabled mimicking ADEs as defined by some [15], [19]. We believe our ADE categorisation improved the understanding of the nature of ADEs and the respondents’ capability to report ADEs, but is was not faultless. The interpretation of “*normal dose*” to differentiate ADRs and intoxications was somewhat ambiguous, and combining the two categories should be considered in future studies. We purposefully separated drug dependence from ADRs, because dependence may be associated with too high a dose. Drug dependence with normal dose may, however, also fall under the definition for ADRs. We purposefully also classified morbiditeis due to drug interactions as ADRs or STEs, according to the health consequence rather than the cause, although some consider all interactions as ADRs. Even though we did not assess the preventability of morbidities due to drug-related untreated indications, we considered prior to conducting the study that such ADEs may or may not be preventable. In hindsight, all such cases could be considered preventable, as an error has occurred if a needed therapy was not initiated. We suggest future studies to further investigate improving the categorisation of all and preventable ADEs, as categorisation appears useful for conceptualising this heterogeneous group of events.

As our data were restricted to a mailed survey, we could not assess the causality or perceived preventability of the reported ADEs against clinical data, nor further interview the respondents. We did ask the respondents to describe the preventable ADEs, which will be reported in a separate article. Although we assessed the questions on ADEs and preventability for face and content validity, some respondents misinterpreted them. Further validation of the survey instrument would have improved our study, and is recommended for future studies. As our study focused on the respondents’ experiences, few ADEs and preventable ADEs were excluded during coding. Therefore, the coding was less likely to influence our results than the recall and reporting biases. Due to these limitations in our case detection, our results must be interpreted with caution and further studies are needed to confirm our findings.

Our respondents were more representative of the general population than in previous studies, but the non-response bias limits the generalisability of our findings to all adults in Sweden. Although our 51% response rate was lower than we anticipated, based on a survey on ADRs to the Swedish general public in 2004 [13], it was in line with the declining response rates in Sweden. For example, the response rate to the National Survey of Public Health has declined from 61% in 2004 to 52% in 2009 [81]. Apart from this trend, our response rate was probably negatively affected by the length of the survey, 21 pages including questions reported elsewhere [41], and the complexity of the research area. In hindsight, fewer questions and further validation could have increased the response rate. The non-response bias probably affected ADE prevalences differently by age. Young non-respondents were probably relatively healthy and may have had fewer ADEs than respondents, possibly resulting in overestimating the ADE prevalence. Among the elderly, however, respondents may have been healthier [49], and less commonly hospitalised or institutionalised, and probably suffered from fewer ADEs than elderly non-respondents, resulting in underestimating the ADE prevalence. Most ADEs were experienced without healthcare encounters and thus probably occurred in the community, but we were unable to assess the origin of them. As we surveyed prevalent ADEs, some ADEs experienced in the community during the study period may have debuted previously in inpatient care, and vice versa. Even though these limitations probably influenced the nature of the reported ADEs and our inability to detect prevalence differences between the age groups, our study still demonstrates that ADEs are a considerable health concern in the community in Sweden, across age groups. Although the prevalence and pattern of ADEs vary depending on the patient population, settings, ADE definitions, and methods [4], the burden of ADEs is also likely to be significant in other geographical areas.

Finally, our ADE prevalences among all respondents were fairly robust for using respondents with self-reported drug use as the denominator. When respondents with healthcare encounters were used as the denominator, the prevalences of ADRs and STEs increased markedly, suggesting that persons with ADRs and STEs visit healthcare more than persons with the other ADEs.

## Conclusions

One fifth of the adult general public reported ADEs during the past month, with our ADE definition, indicating that ADEs are a significant disease burden in all age groups and beyond hospitals. Thus, ADEs experienced by the public must be considered in the prevention of ADEs. Prevalence and associated drugs varied between the ADE categories, suggesting that categorising ADEs enriches characterising ADEs. For developing prevention strategies, the underlying causes of such ADEs should increasingly be investigated. Although there are many solutions to reduce ADEs, the high burden of ADEs and preventable ADEs from widely used drugs across care settings supports, in our view, redesigning a safer healthcare system to adequately tackle the problem.

## Supporting Information

Table S1 Survey questions on ADEs designed for laymen based on pre-defined definitions for ADE categories.
(DOCX)

Table S2 Drug classes and drugs associated to self-reported adverse drug events (ADEs), ordered according to the most commonly dispensed drugs to all respondents.
(DOCX)

Table S3 Organ systems^a^ and symptoms^b^ affected by all and preventable self-reported adverse drug reactions (ADRs), with reported drugs^c^.
(DOCX)

## References

[1] World Health Organization (2009) WHO patient safety research - Better knowledge for safer care. France: World Health Organization.

[2] Ministry of Health and Social Affairs (2011) National medication strategy [in Swedish]. Stockholm: Ministry of Health and Social Affairs.

[3] Bates DW, Cullen DJ, Laird N, Petersen LA, Small SD, et al (1995) Incidence of adverse drug events and potential adverse drug events: Implications for prevention. ADE prevention study group. JAMA 274: 29–34. 7791255

[4] Leendertse AJ, Visser D, Egberts AC, van den Bemt PM (2010) The relationship between study characteristics and the prevalence of medication-related hospitalizations: A literature review and novel analysis. Drug Saf 33: 233–244 10.2165/11319030-000000000-00000. 20158287 10.2165/11319030-000000000-00000

[5] Aronson JK, Ferner RE (2005) Clarification of terminology in drug safety. Drug Saf 28: 851–870. 16180936 10.2165/00002018-200528100-00003

[6] Kanjanarat P, Winterstein AG, Johns TE, Hatton RC, Gonzalez-Rothi R, et al (2003) Nature of preventable adverse drug events in hospitals: A literature review. Am J Health Syst Pharm 60: 1750–1759. 14503111 10.1093/ajhp/60.17.1750

[7] Thomsen LA, Winterstein AG, Sondergaard B, Haugbolle LS, Melander A (2007) Systematic review of the incidence and characteristics of preventable adverse drug events in ambulatory care. Ann Pharmacother 41: 1411–1426 10.1345/aph.1H658. 17666582 10.1345/aph.1H658

[8] Winterstein AG, Sauer BC, Hepler CD, Poole C (2002) Preventable drug-related hospital admissions. Ann Pharmacother 36: 1238–1248. 12086559 10.1345/aph.1A225

[9] Hakkarainen KM, Hedna K, Petzold M, Hägg S (2012) Percentage of patients with preventable adverse drug reactions and preventability of adverse drug reactions - A meta-analysis. PLoS ONE 7: e33236 10.1371/journal.pone.0033236. 22438900 10.1371/journal.pone.0033236 PMC3305295

[10] Krahenbuhl-Melcher A, Schlienger R, Lampert M, Haschke M, Drewe J, et al (2007) Drug-related problems in hospitals: A review of the recent literature. Drug Saf 30: 379–407. 17472418 10.2165/00002018-200730050-00003

[11] Cano FG, Rozenfeld S (2009) Adverse drug events in hospitals: A systematic review. Cad Saude Publica 25 Suppl 3S360–72. 20027385 10.1590/s0102-311x2009001500003

[12] Weissman JS, Schneider EC, Weingart SN, Epstein AM, David-Kasdan J, et al (2008) Comparing patient-reported hospital adverse events with medical record review: Do patients know something that hospitals do not? Ann Intern Med 149: 100–108. 18626049 10.7326/0003-4819-149-2-200807150-00006

[13] Isacson D, Johansson L, Bingefors K (2008) Nationwide survey of subjectively reported adverse drug reactions in Sweden. Ann Pharmacother 42: 347–353 10.1345/aph.1K488. 18303136 10.1345/aph.1K488

[14] Weingart SN, Gandhi TK, Seger AC, Seger DL, Borus J, et al (2005) Patient-reported medication symptoms in primary care. Arch Intern Med 165: 234–240 10.1001/archinte.165.2.234. 15668373 10.1001/archinte.165.2.234

[15] Gandhi TK, Weingart SN, Borus J, Seger AC, Peterson J, et al (2003) Adverse drug events in ambulatory care. N Engl J Med 348: 1556–1564 10.1056/NEJMsa020703. 12700376 10.1056/NEJMsa020703

[16] Chrischilles EA, Segar ET, Wallace RB (1992) Self-reported adverse drug reactions and related resource use. A study of community-dwelling persons 65 years of age and older. Ann Intern Med 117: 634–640. 1530194 10.7326/0003-4819-117-8-634

[17] Hutchinson TA, Flegel KM, Kramer MS, Leduc DG, Kong HH (1986) Frequency, severity and risk factors for adverse drug reactions in adult out-patients: A prospective study. J Chronic Dis 39: 533–542. 3722316 10.1016/0021-9681(86)90198-0

[18] Oladimeji O, Farris KB, Urmie JG, Doucette WR (2008) Risk factors for self-reported adverse drug events among medicare enrollees. Ann Pharmacother 42: 53–61 10.1345/aph.1K073. 18029427 10.1345/aph.1K073

[19] Gandhi TK, Burstin HR, Cook EF, Puopolo AL, Haas JS, et al (2000) Drug complications in outpatients. J Gen Intern Med 15: 149–154. 10718894 10.1046/j.1525-1497.2000.04199.x PMC1495358

[20] Green JL, Hawley JN, Rask KJ (2007) Is the number of prescribing physicians an independent risk factor for adverse drug events in an elderly outpatient population? Am J Geriatr Pharmacother 5: 31–39. 17608245 10.1016/j.amjopharm.2007.03.004

[21] Chrischilles E, Rubenstein L, Van Gilder R, Voelker M, Wright K, et al (2007) Risk factors for adverse drug events in older adults with mobility limitations in the community setting. J Am Geriatr Soc 55: 29–34 10.1111/j.1532–5415.2006.01034.x. 17233682 10.1111/j.1532-5415.2006.01034.x

[22] Shiyanbola OO, Farris KB (2010) Concerns and beliefs about medicines and inappropriate medications: An internet-based survey on risk factors for self-reported adverse drug events among older adults. Am J Geriatr Pharmacother 8: 245–257 10.1016/j.amjopharm.2010.06.002. 20624614 10.1016/j.amjopharm.2010.06.002

[23] Cuijpers P (2011) The patient perspective in research on major depression. BMC Psychiatry 11: 89 10.1186/1471-244X-11-89. 21592336 10.1186/1471-244X-11-89 PMC3112082

[24] Gibson PG, McDonald VM, Marks GB (2010) Asthma in older adults. Lancet 376: 803–813 10.1016/S0140-6736(10)61087-2. 20816547 10.1016/S0140-6736(10)61087-2

[25] Zorba Paster R (2010) Chronic pain management issues in the primary care setting and the utility of long-acting opioids. Expert Opin Pharmacother 11: 1823–1833 10.1517/14656566.2010.491510. 20629606 10.1517/14656566.2010.491510

[26] Al-Olah YH, Al Thiab KM (2008) Admissions through the emergency department due to drug-related problems. Ann Saudi Med 28: 426–429. 19011316 10.5144/0256-4947.2008.426 PMC6074252

[27] Compton WM, Thomas YF, Stinson FS, Grant BF (2007) Prevalence, correlates, disability, and comorbidity of DSM-IV drug abuse and dependence in the united states: Results from the national epidemiologic survey on alcohol and related conditions. Arch Gen Psychiatry 64: 566–576 10.1001/archpsyc.64.5.566. 17485608 10.1001/archpsyc.64.5.566

[28] Jonsson AK, Spigset O, Tjaderborn M, Druid H, Hägg S (2009) Fatal drug poisonings in a Swedish general population. BMC Clin Pharmacol 9: 7 10.1186/1472-6904-9-7. 19397805 10.1186/1472-6904-9-7 PMC2679715

[29] Zargarzadeh AH, Emami MH, Hosseini F (2007) Drug-related hospital admissions in a generic pharmaceutical system. Clin Exp Pharmacol Physiol 34: 494–498 10.1111/j.1440-1681.2007.04600.x. 17439421 10.1111/j.1440-1681.2007.04600.x

[30] Raschetti R, Morgutti M, Menniti-Ippolito F, Belisari A, Rossignoli A, et al (1999) Suspected adverse drug events requiring emergency department visits or hospital admissions. Eur J Clin Pharmacol 54: 959–963. 10192758 10.1007/s002280050582

[31] Franceschi A, Tuccori M, Bocci G, Vannozzi F, Di Paolo A, et al (2004) Drug therapeutic failures in emergency department patients. A university hospital experience. Pharmacol Res 49: 85–91. 14597157 10.1016/j.phrs.2003.08.001

[32] Hallas J, Harvald B, Gram LF, Grodum E, Brosen K, et al (1990) Drug related hospital admissions: The role of definitions and intensity of data collection, and the possibility of prevention. J Intern Med 228: 83–90. 2394974 10.1111/j.1365-2796.1990.tb00199.x

[33] Morimoto T, Gandhi TK, Seger AC, Hsieh TC, Bates DW (2004) Adverse drug events and medication errors: Detection and classification methods. Qual Saf Health Care 13: 306–314 10.1136/qhc.13.4.306. 15289635 10.1136/qshc.2004.010611 PMC1743868

[34] Edwards IR, Aronson JK (2000) Adverse drug reactions: Definitions, diagnosis, and management. Lancet 356: 1255–1259 10.1016/S0140-6736(00)02799-9. 11072960 10.1016/S0140-6736(00)02799-9

[35] Goettler M, Schneeweiss S, Hasford J (1997) Adverse drug reaction monitoring - Cost and benefit considerations. Part II: Cost and preventability of adverse drug reactions leading to hospital admission. Pharmacoepidemiol Drug Saf 6 Suppl 3 S79–90: 2–O. 10.1002/(sici)1099-1557(199710)6:3+<s79::aid-pds294>3.3.co;2-f 15073758

[36] World Health Organization (1972) Technical report series. International drug monitoring: The role of national centres. Geneva: World Health Organization. 4625548

[37] Uppsala Monitoring Centre (2013) Glossary of terms used in pharmacovigilance. Uppsala Monitoring Centre.

[38] van Gijssel-Wiersma DG, van den Bemt PM, Walenbergh-van Veen MC (2005) Influence of computerised medication charts on medication errors in a hospital. Drug Saf 28: 1119–1129. 16329714 10.2165/00002018-200528120-00006

[39] Hallas J, Harvald B, Worm J, Beck-Nielsen J, Gram LF, et al (1993) Drug related hospital admissions: Results from an intervention program. Eur J Clin Pharmacol 45: 199–203. 8276041 10.1007/BF00315383

[40] American Psychiatric Association (2000) Diagnostic and statistical manual of mental disorders (DSM-IV). Washington, DC: American Psychiatric Association.

[41] Gyllensten H, Rehnberg C, Jonsson AK, Petzold M, Carlsten A, et al.. (2013) Cost of illness of patient-reported adverse drug events: A population-based cross-sectional survey. BMJ Open 3: 10.1136/bmjopen-2013-002574. Print 2013. 10.1136/bmjopen-2013-002574; 10.1136/bmjopen-2013-002574. 10.1136/bmjopen-2013-002574 PMC3686161 23794552

[42] Wettermark B, Hammar N, Fored CM, Leimanis A, Otterblad Olausson P, et al (2007) The new Swedish Prescribed Drug Register - Opportunities for pharmacoepidemiological research and experience from the first six months. Pharmacoepidemiol Drug Saf 16: 726–735 10.1002/pds.1294. 16897791 10.1002/pds.1294

[43] World Health Organization (2009) Guidelines for ATC classification and DDD assignment 2010. Oslo: WHO Collaborating Centre for Drug Statistics Methodology.

[44] Danielsson M, Talback M (2012) Public health: An overview: Health in Sweden: The national public health report 2012. Chapter 1. Scand J Public Health 40: 6–22 10.1177/1403494812459457; 10.1177/1403494812459457. 10.1177/1403494812459457 23238399

[45] Weitoft GR, Ericsson O, Fastbom J (2012) Prescription drugs: Health in Sweden: The national public health report 2012. Chapter 18. Scand J Public Health 40: 293–304 10.1177/1403494812459623; 10.1177/1403494812459623. 23238416 10.1177/1403494812459623

[46] Medical Dictionary for Regulatory Activities (2011) Introductory guide MedDRA version 14.0, document MSSO-DI-6003-14.0.0. Geneva: Medical Dictionary for Regulatory Activities.

[47] Gurwitz JH, Field TS, Avorn J, McCormick D, Jain S, et al (2000) Incidence and preventability of adverse drug events in nursing homes. Am J Med 109: 87–94. 10967148 10.1016/s0002-9343(00)00451-4

[48] Masotti P, McColl MA, Green M (2010) Adverse events experienced by homecare patients: A scoping review of the literature. Int J Qual Health Care 22: 115–125 10.1093/intqhc/mzq003. 20147333 10.1093/intqhc/mzq003

[49] de Souto Barreto P (2012) Participation bias in postal surveys among older adults: The role played by self-reported health, physical functional decline and frailty. Arch Gerontol Geriatr 55: 592–592 10.1016/j.archger.2012.03.008. 22534027 10.1016/j.archger.2012.03.008

[50] Gyllensten H, Hakkarainen KM, Jonsson AK, Andersson Sundell K, Hägg S, et al (2012) Modelling drug-related morbidity in Sweden using an expert panel of pharmacists. Int J Clin Pharm 34: 538–546 10.1007/s11096-012-9641-3. 22544221 10.1007/s11096-012-9641-3

[51] Hakkarainen KM, Alstrom D, Hägg S, Carlsten A, Gyllensten H (2012) Modelling drug-related morbidity in Sweden using an expert panel of physicians. Eur J Clin Pharmacol 68: 1309–1319 10.1007/s00228-012-1244-3. 22392557 10.1007/s00228-012-1244-3

[52] Swedish National Institute of Public Health (2010) Narcotics use in Sweden [in Swedish]. Östersund: Swedish National Institute of Public Health. 66 p.

[53] Koneri R, Prakasam K, Mishra V, Rajan H (2008) Drug-related hospitalizations at a tertiary level hospital in bangalore: A prospective study. J Clin Diagn Res 2: 736–740.

[54] Nelson KM, Talbert RL (1996) Drug-related hospital admissions. Pharmacotherapy 16: 701–707. 8840382

[55] Hallas J, Gram LF, Grodum E, Damsbo N, Brosen K, et al (1992) Drug related admissions to medical wards: A population based survey. Br J Clin Pharmacol 33: 61–68. 1540492 10.1111/j.1365-2125.1992.tb04001.x PMC1381200

[56] Rogers S, Wilson D, Wan S, Griffin M, Rai G, et al (2009) Medication-related admissions in older people: A cross-sectional, observational study. Drugs Aging 26: 951–961 10.2165/11316750-000000000-00000. 19848440 10.2165/11316750-000000000-00000

[57] Johnson JA, Bootman JL (1995) Drug-related morbidity and mortality. A cost-of-illness model. Arch Intern Med 155: 1949–1956. 7575048

[58] Avery AJ, Anderson C, Bond CM, Fortnum H, Gifford A, et al.. (2011) Evaluation of patient reporting of adverse drug reactions to the UK ‘yellow card scheme’: Literature review, descriptive and qualitative analyses, and questionnaire surveys. Health Technol Assess 15: 1–234, iii–iv. 10.3310/hta15200; 10.3310/hta15200. 10.3310/hta15200 21545758

[59] Pirmohamed M, James S, Meakin S, Green C, Scott AK, et al (2004) Adverse drug reactions as cause of admission to hospital: Prospective analysis of 18 820 patients. BMJ 329: 15–19 10.1136/bmj.329.7456.15. 15231615 10.1136/bmj.329.7456.15 PMC443443

[60] Kongkaew C, Noyce PR, Ashcroft DM (2008) Hospital admissions associated with adverse drug reactions: A systematic review of prospective observational studies. Ann Pharmacother 42: 1017–1025 10.1345/aph.1L037. 18594048 10.1345/aph.1L037

[61] Davies EC, Green CF, Taylor S, Williamson PR, Mottram DR, et al (2009) Adverse drug reactions in hospital in-patients: A prospective analysis of 3695 patient-episodes. PLoS ONE 4: e4439 10.1371/journal.pone.0004439. 19209224 10.1371/journal.pone.0004439 PMC2635959

[62] Wiffen P, Gill M, Edwards J, Moore A (2002) Adverse drug reactions in hospital patients – A systematic review of the prospective and retrospective studies. Bandolier Extra: 1–16.

[63] Millar JS (2001) Consultations owing to adverse drug reactions in a single practice. Br J Gen Pract 51: 130–131. 11217627 PMC1313929

[64] Dequito AB, Mol PG, van Doormaal JE, Zaal RJ, van den Bemt PM, et al (2011) Preventable and non-preventable adverse drug events in hospitalized patients: A prospective chart review in the netherlands. Drug Saf 34: 1089–1100 10.2165/11592030-000000000-00000. 21981436 10.2165/11592030-000000000-00000

[65] Howard RL, Avery AJ, Slavenburg S, Royal S, Pipe G, et al (2007) Which drugs cause preventable admissions to hospital? A systematic review. Br J Clin Pharmacol 63: 136–147 10.1111/j.1365–2125.2006.02698.x. 16803468 10.1111/j.1365-2125.2006.02698.x PMC2000562

[66] Edwards IR (2012) Prevention and pharmacovigilance: What should we do, what can we do? Drug Saf 35: 87–90 10.2165/11630510-000000000-00000. 22242806 10.2165/11630510-000000000-00000

[67] Bates DW, Miller EB, Cullen DJ, Burdick L, Williams L, et al (1999) Patient risk factors for adverse drug events in hospitalized patients. ADE prevention study group. Arch Intern Med 159: 2553–2560. 10573045 10.1001/archinte.159.21.2553

[68] Runciman WB, Edmonds MJ, Pradhan M (2002) Setting priorities for patient safety. Qual Saf Health Care 11: 224–229. 12486985 10.1136/qhc.11.3.224 PMC1743639

[69] Basch E (2010) The missing voice of patients in drug-safety reporting. N Engl J Med 362: 865–869 10.1056/NEJMp0911494; 10.1056/NEJMp0911494. 20220181 10.1056/NEJMp0911494 PMC3031980

[70] Lau PM, Stewart K, Dooley M (2004) The ten most common adverse drug reactions (ADRs) in oncology patients: Do they matter to you? Support Care Cancer 12: 626–633 10.1007/s00520-004-0622-5. 15064936 10.1007/s00520-004-0622-5

[71] Vincent C (2010) Clinical interventions and process improvement. In: Vincent C, editor. Patient Safety. West Sussex: Wiley-Blackwell. 211–229.

[72] Bates DW, Leape LL, Cullen DJ, Laird N, Petersen LA, et al (1998) Effect of computerized physician order entry and a team intervention on prevention of serious medication errors. JAMA 280: 1311–1316. 9794308 10.1001/jama.280.15.1311

[73] Leape LL, Cullen DJ, Clapp MD, Burdick E, Demonaco HJ, et al (1999) Pharmacist participation on physician rounds and adverse drug events in the intensive care unit. JAMA 282: 267–270. 10422996 10.1001/jama.282.3.267

[74] Nanji KC, Rothschild JM, Salzberg C, Keohane CA, Zigmont K, et al (2011) Errors associated with outpatient computerized prescribing systems. J Am Med Inform Assoc 18: 767–773 10.1136/amiajnl-2011-000205; 10.1136/amiajnl-2011-000205. 21715428 10.1136/amiajnl-2011-000205 PMC3197998

[75] Patterson ES, Cook RI, Render ML (2002) Improving patient safety by identifying side effects from introducing bar coding in medication administration. J Am Med Inform Assoc 9: 540–553. 12223506 10.1197/jamia.M1061 PMC346641

[76] Woods DD (2000) Behind human error: Human factors research to improve patient safety. National Summit on Medical Errors and Patient Safety Research, Quality Interagency Coordination Task Force and Agency for Healthcare Research and Quality.

[77] Nolan TW (2000) System changes to improve patient safety. BMJ 320: 771–773. 10720364 10.1136/bmj.320.7237.771 PMC1117771

[78] Vincent C (2010) High performing healthcare systems. In: Vincent C, editor. Patient Safety. West Sussex: Wiley-Blackwell. 390–404.

[79] Leape LL (2006) System analysis and redesign: The foundation of medical error prevention. In: Cohen MR, editor. Medication errors. Washington DC: American Pharmacists’ Association. 3–14.

[80] Hakkarainen KM, Andersson Sundell K, Petzold M, Hägg S (2012) Methods for assessing the preventability of adverse drug events: A systematic review. Drug Saf 35: 105–126 10.2165/11596570-000000000-00000. 22201475 10.2165/11596570-000000000-00000

[81] Boström G (2009) The impact of non-response bias in public health surveys [in Swedish]. Östersund: National Institute of Public Health. 2 p.

