# Supplementary material for: Prevalence and Perceived Preventability of Self-Reported Adverse Drug Events – A Population-Based Survey of 7099 Adults
Source: PLoS One. 2013 Sep 4;8(9):e73166. doi: 10.1371/journal.pone.0073166 (PMC3762841; doi:10.1371/journal.pone.0073166)
Supplement: Table S1 — Survey questions on ADEs designed for laymen based on pre-defined definitions for ADE categories. (DOCX) [file pone.0073166.s001.docx]

**Table S1.** Survey questions on ADEs designed for laymen based on pre-defined definitions for ADE categories.

| **Definition for ADE category prior to survey design** | **Survey question on ADE category (yes/no)** | **If yes, further specification** | **Reasons for exclusion from results** |
| --- | --- | --- | --- |
| Adverse drug reaction: a response to a drug which is noxious and unintended, and which occurs at doses normally used in man [36,37] | Have you experienced a/some side effects of drugs^a^ during the past 30 days? | Please describe the symptoms and drugs for the maximum of three side effects. If you have experienced more than three side effects, please specify the side effects that influenced you the most. You can specify multiple drugs for one side effect or the same drug for multiple side effects. [for each three adverse reaction, one response box for the symptoms and one response box for the associated drugs] | - Given treatment not drug (n=1)  - The same ADE multiple times (n=2)  - General description of care without ADE (n=15)  -Answered “*Don’t know*” in response box (n=1) |
| Drug intoxication from overdose: a noxious, intended or unintended drug reaction that occurs at higher doses than normally used in man for prophylaxis, diagnosis or treatment; the intention for administrating the drug(s) may or may not be therapeutic^b^ | Have you during the past 30 days consciously or unconsciously taken or got too much of a drug^a^ resulting in a poisoning? A poisoning is a strong reaction of too high a dose of one or more drugs. | Please specify one or more drugs that you a got a poisoning from. [for each three intoxications, one response box for the associated drugs] | - |
| Drug dependence: a maladaptive pattern of use of an addictive drug leading to clinically significant impairment or distress, modified from [40]^c^ | Some drugs^a^ can cause dependence, for example that you can not discontinue drug use even if you wanted to. Do you perceive that you have had drug dependence during the past 30 days? | Please specify one or more drugs that you have had drug dependence on. [for each three dependences, one response box for the dependence drug] | - Given treatment not drug (n=1)  - Specified drug not addictive^d^ (n=567)  - The same ADE multiple times (n=1)  - General description of care without ADE (n=2) |
| Sub-therapeutic effect of drug therapy: an absence of therapeutic response that could be linked causally either to (prescribed) dose that was too low, to drug non-compliance, recent dose reduction/discontinuation or inadequate monitoring, or to improper drug selection. Sub-optimal therapeutic effect can also occur when the treatment has been rational (e.g. first line treatment or best available medicines were not effective enough). (modified from [32]) | Have you during the past 30 days experienced insufficient effect from your drugs^a^? | Please specify the maximum of three the drugs. If the effect of more than three drugs was insufficient, please specify the drugs whose insufficient effect influenced you the most. Specify drugs even if you expected insufficient effect. [for each three sub-therapeutic effect, one response box for the associated drug] | - Given treatment not drug (n=1)  - The same ADE multiple times (n=1)  - Describes an ADR (n=1)  - General description of care without ADE (n=2)  -Answered “*No*” or “*Don’t know*” in response box (n=2) |
| Morbidity due to drug-related untreated indication: a clinical condition that under normal circumstances requires pharmacological therapy but the person is not receiving any drug therapy for the condition^d^ | The next question is on if you have had symptoms for which you perceive a drug therapy would have been required but you did not get or use a drug for. Have you during the past 30 days experienced symptoms that you perceive you should have got or taken a drug^a^ for? Please specify conditions that you have sought care for and conditions that you haven’t sought care for. | Please specify the symptoms. [for each three morbidity due to drug-related untreated indication, one response box for the symptoms] | - The same ADE multiple times (n=4)  - Describes drug use (n=13)  - General description of care without ADE (n=13) |

^a^The inclusion of prescription, non-prescription and herbal drugs specified earlier in the questionnaire.

^b^Developed by the research group, separating intoxications from adverse drug reactions.

^c^Specification for addictive drugs added by the research group, because the Swedish language does not differentiate “dependence of” and dependent on”, resulting in reporting of drugs people depend on, such as insulin. Addictive drugs included those classified as narcotics in the Swedish Medicines Information Engine (FASS), and five additional drugs with evidence on addictive properties: caffeine, codeine, nicotine, pregabalin, and dextropropoxyphene.

^d^Developed by the research group.
