# Supplementary material for: Prevalence and Perceived Preventability of Self-Reported Adverse Drug Events – A Population-Based Survey of 7099 Adults
Source: PLoS One. 2013 Sep 4;8(9):e73166. doi: 10.1371/journal.pone.0073166 (PMC3762841; doi:10.1371/journal.pone.0073166)
Supplement: Table S2 — Drug classes and drugs associated to self-reported adverse drug events (ADEs), ordered according to the most commonly dispensed drugs to all respondents. (DOCX) [file pone.0073166.s002.docx]

**Table S2.** Drug classes and drugs associated to self-reported adverse drug events (ADEs), ordered according to the most commonly dispensed drugs to all respondents.

| **Drug class**^a^ | **Dispensed to all respondents**^c^ **(n=7099), n (%)**^a,b^ | **ADRs (n=847), n (%)**^a,b^ | **Sub-therapeutic effects of drug therapy (n=745), n (%)**^a,b^ | **Drug dependence (n=174), n (%)**^a,b^ | **Drug intoxications from overdose (n=20), n (%)**^a,b^ |
| --- | --- | --- | --- | --- | --- |
| **Cardiovascular system** | **2093 (29.5)** | **163 (19.2)** | **52 (7.0)** | **0 (0)** | **6 (30.0)** |
| Agents acting on the renin-angiotensin system | **1104 (15.6)**: enalapril 442 (40) | **37 (4.4)**: enalapril 18 (49), losartan 11 (30) | **16 (2.2)**: enalapril 5 (31), losartan 5 (31) | **-** | **2 (10.0)**: telmisartan 1 (50), aliskiren 1 (50) |
| Beta blocking agents | **983 (13.8)**: metoprolol 527 (54), atenolol 221 (22) | **33 (3.9)**: metoprolol 17 (52), bisoprolol 9 (27) | **14 (1.9)**: bisoprolol 5 (36), metoprolol 4 (29) | **-** | **1 (5.0)**: metoprolol 1 (100) |
| Lipid modifying agents | **891 (12.6)**: simvastatin 734 (82) | **28 (3.3)**: simvastatin 18 (64) | **-** | **-** | **1 (5.0)**: simvastatin 1 (100) |
| Diuretics | **659 (9.3)**: furosemide 253 (38), bendroflumethiazide 149 (23) | **15 (1.8)**: bendroflumethiazide 5 (33), furosemide 4 (27), eplerenone 4 (27) | **8 (1.1)**: hydrochlorothiazide 2 (25) | **-** | **-** |
| Calcium channel blockers | **586 (8.3)**: amlodipine 279 (48), felodipine 250 (43) | **21 (2.5)**: felodipine 10 (48), amlodipine 6 (29), verapamil 5 (24) | **-** | **-** | **1 (5.0)**: amlodipine 1 (100) |
| Cardiac therapy | **284 (4.0)**: glyceryl trinitrate 113 (40), isosorbide mononitrate 84 (30) | **-** | **-** | **-** | **1 (5.0)**: isosorbide mononitrate 1 (100) |
| Vasoprotectives | **71 (1.0)**: prednisolone 28 (39), hydrocortisone 26 (37) | **-** | **-** | **-** | **-** |
| **Nervous system** | **1700 (23.9)** | **281 (33.2)** | **238 (32.0)** | **163 (93.7)** | **10 (50.0)** |
| Analgesics | **890 (12.5)**: paracetamol 512 (57.5) | **63 (7.4)**: tramadol 16 (25) | **124 (16.6)**: paracetamol 74 (60) | **37 (21.3)**: tramadol 12 (32), acetylsalicylic acid combinations excl. psycholeptics^f^ 9 (24) | **4 (20.0)**: codeine combinations excl. psycholeptics 1 (25), tramadol 1 (25), acetylsalicylic acid combinations excl. psycholeptics 1 (25), paracetamol 1 (25) |
| Psycholeptics | **756 (10.6)** | **45 (5.3)** | **39 (5.2)** | **116 (66.7)** | **5 (25.0)** |
| *Hypnotics and sedatives* | **499 (7.0)**: zopiclone 214 (43), zolpidem 178 (36) | **17 (2.0)**: zopiclone 9 (53), zolpidem 4 (24) | **25 (3.4)**: zopiclone 8 (32), zolpidem 6 (24) | **93 (53.5)**: zopiclone 33 (36), zolpidem 30 (32) | **3 (15.0)**: zopiclone 1 (33), zolpidem 1 (33) |
| *Anxiolytics* | **212 (3.0)**: oxazepam 94 (44), hydroxyzine 74 (35) | **12 (1.4)**: diazepam 4 (33), oxazepam 3 (25), hydroxyzine 3 (25) | **8 (1.1)**: hydroxyzine 4 (50), oxazepam 2 (25) | **22 (12.6)**: oxazepam 14 (64) | **1 (5.0)**: oxazepam 1 (100) |
| *Antipsychotics incl. lithium* | **-** | **16 (1.9)**: lithium 6 (38) | **-** | **-** | **1 (5.0)**: perphenazine 1 (100) |
| Psychoanaleptics | **551 (7.8)** | **140 (16.5)** | **48 (6.4)** | **3 (1.7)** | **1 (5.0)** |
| *Antidepressants* | **522 (7.4)**: citalopram 152 (29), sertraline 108 (21) | **130 (15.4)**: sertraline 30 (23), citalopram 29 (22) | **43 (5.8)** | **-** | **1 (5.0)**: fluoxetine 1 (100) |
| *Psychostimulants, agents used for*  *ADHD and nootropics* | **-** | **-** | **-** | **3 (1.7**): caffeine 1 (33) | **-** |
| Antiepileptics | **145 (2.0)**: gabapentin 32 (22), carbamazepine 29 (20) | **21 (2.4)**: pregabalin 6 (29), carbamazepine 5 (24), gabapentin 5 (24) | **11 (1.5)**: pregabalin 4 (36) | **-** | **-** |
| Anti-parkinson drugs | **-** | **-** | **13 (1.7)**: pramipexole 4 (31), levodopa and decarboxylase inhibitor 3 (23) | **-** | **-** |
| Other nervous system drugs | **-** | **-** | - | **6 (3.5)**: nicotine 5 (83) | **-** |
| **Alimentary tract and metabolism** | **1501 (21.1)** | **34 (4.0)** | **70 (9.4)** | **0 (0)** | **0 (0)** |
| Drugs for acid related disorders | **660 (9.3)**: omeprazole 478 (72), esomeprazole 85 (13) | **10 (1.2)**: omeprazol 7 (70) | **33 (4.3)**: omeprazol 17 (52) | **-** | **-** |
| Drugs used in diabetes | **352 (5.0)**: metformin 151 (43) | **11 (1.3)**: metformin 8 (73) | **15 (2.1)**: metformin 4 (27), insulin human 3 (20) | **-** | **-** |
| Drugs for constipation | **309 (4.4)**: sterculia 63 (20), macrogol combinations 93 (30) | **-** | **-** | **-** | **-** |
| Mineral supplements | **301 (4.2)**: Calcium, combinations with vitamin D and/or other drugs 219 (73) | **-** | **-** | **-** | **-** |
| Stomatological preparations | **180 (2.5)**: sodium fluoride 150 (83) | **-** | **-** | **-** | **-** |
| Antidiarrheals, intestinal antiinflammatory/antiinfective agents | **96 (1.4)**: loperamide 44 (46) | **9 (1.1)**: loperamide 3 (33), loperamide combinations 2 (22), sulfasalazine 2 (22), mesalazine 2 (22) | **-** | **-** | **-** |
| Vitamins | **91 (1.3)**: Vitamin B-complex plain 42 (46) | **-** | **-** | **-** | **-** |
| **Blood and blood forming organs** | **1219 (17.2)** | **24 (2.8)** | **10 (1.3)** | **0 (0)** | **2 (10.0)** |
| Antithrombotic agents | **975 (13.7)**: acetylsalicylic acid 683 (70) | **16 (1.9)**: warfarin 7 (44), acetylsalicylic acid 7 (44) | **-** | **-** | **2 (10.0)**: warfarin 1 (50), acetylsalicylic acid 1 (50) |
| Antianemic preparations | **396 (5.6)**: cyanocobalamin 227 (57), folic acid 91 (23) | **-** | **-** | **-** | **-** |
| **Genito urinary system and sex hormones** | **1109 (15.6)** | **53 (6.3)** | **24 (3.2)** | **0 (0)** | **0 (0)** |
| Sex hormones and modulators of the genital system | **826 (11.6)**: estradiol 272 (33) | **39 (4.6)** | **8 (1.1)**: estradiol 2 (25) | **-** | **-** |
| Urologicals | **257 (3.6)**: alfuzosin 63 (25) | **13 (1.5)**: tadalafil 4 (31), tolterodine 3 (23), darifenacin 3 (23) | **13 (1.7)**: finasteride 4 (31) | **-** | **-** |
| **Respiratory system** | **1110 (15.6)** | **56 (6.6)** | **73 (9.8)** | **0 (0)** | **0 (0)** |
| Drugs for obstructive airways disease | **424 (6.0)**: terbutaline 88 (21) | **20 (2.4)**: terbutaline 10 (50), formoterol and other drugs for obstructive airway diseases 4 (20) | **17 (2.3)** | **-** | **-** |
| Cough and cold preparations | **404 (5.7)**: opium derivatives and expectorants 167 (41), acetylcysteine 126 (31), mucolytics combinations 88 (22) | **14 (1.7)**: opium derivatives and expectorants 8 (57) | **22 (3.0)** | **-** | **-** |
| Nasal preparations | **338 (4.8)**: mometasone 191 (57) | **14 (1.5)**: mometasone 3 (23) | **18 (2.4)**: mometasone 7 (39) | **-** | **-** |
| Antihistamines for systemic use | **300 (4.2)**: cetirizine 84 (28), desloratadine 75 (25) | **-** | **9 (1.2)**: cetirizine 3 (33), loratadine 2 (22) | **-** | **-** |
| **Antiinfectives for systemic use** | **1077 (15.2)** | **42 (5.0)** | **24 (3.2)** | **0 (0)** | **2 (10.0)** |
| Antibacterials for systemic use | **974 (13.7)**: phenoxymethylpenicillin 288 (30) | **35 (4.1)**: phenoxymethylpenicillin 9 (26) | **23 (3.1)** | **-** | **-** |
| Antivirals for systemic use | **72 (1.0)**: acyclovir 32 (44), valaciclovir 32 (44) | **-** | **-** | **-** | **-** |
| Immune sera and immunoglubulins | **-** | **-** | **-** | **-** | **~~-~~** |
| Vaccines | **-** | **-** | **-** | **-** | **1 (5.0)** |
| **Musculo-skeletal system** | **895 (12.6)** | **61 (7.2)** | **111 (14.9)** | **1 (0.6)** | **1 (5.0)** |
| Antiinflammatory and antireumatic products | **697 (9.8)**: diclofenac 354 (51) | **57 (6.7)**: diclofenac 21 (37), naproxen 15 (26), ibuprofen 13 (23) | **104 (14.0)**: ibuprofen 42 (40), diclofenac 35 (34) | **-** | **1 (5.0)**: ibuprofen 1 (100) |
| Drugs for treatment of bone diseases | **103 (1.5)**: alendronic acid 69 (67), risedronic acid 24 (23) | **-** | **-** | **-** | **-** |
| Antigout preparations | **83 (1.2)**: allopurinol 76 (92) | **-** | **-** | **-** | **-** |
| **Systemic hormonal preparations**^d^ | **667 (9.4)** | **46 (5.4)** | **14 (1.9)** | **0 (0)** | **0 (0)** |
| Thyroid therapy | **399 (5.6)**: levothyroxine sodium 394 (99) | **11 (1.3)**: levothyroxine 8 (73), liothyronine sodium 3 (27) | **8 (1.1)**: levothyroxine 7 (88) | **-** | **-** |
| Corticosteroids for systemic use | **293 (4.1)**: prednisolone 166 (57), betamethasone 110 (38) | **32 (3.8)**: prednisolone 14 (44) | **-** | **-** | **-** |
| **Dermatologicals** | **633 (8.9)** | **10 (1.2)** | **42 (5.6)** | **0 (0)** | **0 (0)** |
| Corticosteroids dermatological preparations | **305 (4.3)**: betamethasone 87 (29), mometasone 82 (27) | **-** | **19 (2.6)** | **-** | **-** |
| Emollients and protectives | **206 (2.9)**: carbamide 112 (54), other emollients and protectives 85 (41) | **-** | **-** | **-** | **-** |
| Antifungals for dermatological use | **140 (2.0)**: Imidazole and triazole derivatives combinations 68 (49) | **-** | **-** | **-** | **-** |
| **Sensory organs** | **554 (7.8)** | **5 (0.6)** | **5 (0.7)** | **0 (0)** | **0 (0)** |
| Ophthalmologicals | **460 (6.5)** | **-** | **-** | **-** | **-** |
| Ophthalmologicals and otological preparations | **97 (1.4)**: hydrocortisone and antiinfectives 97 (100) | **-** | **-** | **-** | **-** |
| **Antineoplastic and immunomodulating agents** | **178 (2.5)** | **73 (8.6)** | **12 (1.6)** | **0 (0)** | **0 (0)** |
| Endocrine therapy | **76 (1.1)**: tamoxifen 32 (42), bicalutamide 20 (26) | **30 (3.5)**: tamoxifen 17 (57), anastrozole 7 (23) |  |  |  |
| Immunosuppressants |  | **21 (2.5)**: methotrexate 13 (62) |  |  |  |
| Antineoplastic agents |  | **15 (1.8)**: temozolomide 3 (20), doxorubicin 3 (20) |  |  |  |
| **No dispensed drugs during the past 6 months** | **2417 (34.0)** | NA | NA | NA | NA |
| **ATC not available**^e^ | NA | **115 (11.9)** | **69 (9.3)** | **10 (5.8)** | **0 (0)** |
| Complementary medicine | NA | **2 (0.2)** | **17 (2.3)** | **0 (0)** | **0 (0)** |

ADE = adverse drug event; ADR = adverse drug reaction; ATC = Anatomical Therapeutic Chemical; NA = not applicable.

^a^Categorised according to the Anatomical Therapeutic Chemical (ATC) Classification System [43] main groups (1^st^ level), and pharmacological subgroups (3^rd^ level) representing >1% of the ADE category. Psycholeptics and psychoanaleptics were also categorised into the 4^th^ level drug classes.

^b^Anatomical Therapeutic Chemical (ATC) Classification System [43] chemical substances (5^th^ level) that represent ≥20% of the given pharmacological subgroup.

^c^Dispensed drugs from the Swedish Prescribed Drug Register, including all drugs with a unique the Anatomical Therapeutic Chemical (ATC) Classification System [43] code for each respondent, dispensed from six months before the survey return date until the return date.

^d^Excluding sex hormones and insulins.

^e^Drug missing or unclear, or complementary medicine.

^f^Includes caffeine combinations.
