# Supplementary material for: Prevalence and Perceived Preventability of Self-Reported Adverse Drug Events – A Population-Based Survey of 7099 Adults
Source: PLoS One. 2013 Sep 4;8(9):e73166. doi: 10.1371/journal.pone.0073166 (PMC3762841; doi:10.1371/journal.pone.0073166)
Supplement: Table S3 — Organ systemsa and symptomsb affected by all and preventable self-reported adverse drug reactions (ADRs), with reported drugsc. (DOCX) [file pone.0073166.s003.docx]

**Table S3.** Organ systems^a^ and symptoms^b^ affected by all and preventable self-reported adverse drug reactions (ADRs), with reported drugs^c^.

| **Organ systems**^a^ **and symptoms**^b^ | **ADRs (n=847) n (%)** | **Preventable ADRs (n=139) n (%)** | **Drugs**^c^ **associated with the ADR symptom**^b^ **≥2 times** |
| --- | --- | --- | --- |
| **Gastrointestinal disorders** | **228 (26.9)** | **28 (20.1)** |  |
| Nausea | 55 (6.5) | 9 (6.5) | Methotrexate 4, citalopram 2, codeine combinations excl. psycholeptics 2, diclofenac 2, naproxen 2, oxycodone 2, pregabalin 2 |
| Diarrhoea | 32 (3.8) | 2 (1.4) | Phenoxymethylpenicillin 4, diclofenac 3, metformin 3, flucloxacillin 2, sertraline 2 |
| Dry mouth | 30 (3.5) | - | Sertraline 4, tramadol 3, citalopram 2, tolterodine 2, zopiclone 2 |
| Abdominal pain upper | 28 (3.3) | 4 (2.9) | Diclofenac 3, methotrexate 2, simvastatin 2 |
| Gastric disorder | 24 (2.8) | 4 (2.9) | Diclofenac 3, naproxen 3, metformin 2 |
| Constipation | 22 (2.6) | 4 (2.9) | Codeine, combinations excl. psycholeptics 2, ferrous glycine sulfate 2, omeprazole 2, opium derivatives and expectorants 2, tramadol 2 |
| Vomiting | - | 2 (1.4) | - |
| **General disorders and administration site conditions** | **148 (17.5)** | **17 (12.2)** |  |
| Fatigue | 94 (11.1) | 8 (5.8) | Cetirizine 4, codeine, combinations excl. psycholeptics 4, anastrozole 3, diclofenac 3, ibuprofen 3, sumatriptan 3, zopiclone 3, acetylsalicylic acid 2, amitriptyline2, carbamazepine 2, hydroxyzine 2, levetiracetam 2, lithium 2, loperamide combinations 2, mirtazapine 2, paracetamol 2, pregabalin 2, sertraline 2, topiramate 2, tramadol 2, venlafaxine2 |
| Pain | - | 4 (2.9) | - |
| Hyperhidrosis | - | 3 (2.2) | Tamoxifen 5, sertraline 3, citalopram 2, duloxetine 2 |
| **Nervous system disorders** | **98 (11.9)** | **17 (12.2)** |  |
| Dizziness | 33 (3.9) | 8 (5.8) | Citalopram 3, sertraline 3 |
| Headache | 23 (2.7) | 3 (2.2) | Methotrexate 3, citalopram 2, levonorgestrel and estrogen 2 |
| Tremor | 12 (1.4) | - | Terbutaline 5 |
| Hypoaesthesia |  | 2 (1.4) |  |
| **Psychiatric disorders** | **97 (11.5)** | **20 (14.4)** |  |
| Anxiety | 20 (2.4) | 3 (2.2) | Citalopram 3, levothyroxine 2, methylphenidate 2 |
| Insomnia | 15 (1.8) | 4 (2.9) | - |
| Depressed mood | 14 (1.7) | 5 3.6) | - |
| **Skin and subcutaneous tissue disorders** | **43 (5.1)** | **12 (8.6)** |  |
| Pruritus | 10 (1.3) | 3 (2.2) | - |
| Alopecia | 9 (1.1) | - | Methotrexate 2 |
| Rash | - | 3 (2.2) | Simvastatin 2 |
| **Musculoskeletal and connective tissue disorders** | **43 (5.1)** | **10 (7.2)** |  |
| Muscle spasms | 11 (1.3) | 3 (2.2) | - |
| **Cardiac disorders** | **32 (3.8)** | **7 (5.0)** |  |
| Palpitations | 10 (1.2) | - | Terbutaline 3, formoterol 2 |
| Dizziness | 9 (1.1) | - | Enalapril 2 |
| **Respiratory, thoracic and mediastinal disorders** | **31 (3.7)** | **5 (3.6)** |  |
| Epistaxis | - | 2 (1.4) | Mometasone 2 |
| **Reproductive system and breast disorders** | **29 (3.4)** | **5 (3.6)** |  |
| Erectile dysfunction | - | 2 (1.4) | Enalapril 2 |
| Metrorrhagia | - | 2 (1.4) | **-** |
| **Vascular disorders** | **17 (2.0)** | **6 (4.3)** |  |
| **Investigations** | **14 (1.7)** | **2 (1.4)** |  |
| Weight increased | 14 (1.7) | 2 (1.4) | Escitalopram 2 |
| **Renal and urinary disorders** | **14 (1.6)** | - |  |
| **Eye disorders** | **10 (1.2)** | **3 (2.2)** |  |
| **Metabolism and nutrition disorders** | **9 (1.1)** | **2 (1.4)** |  |
| Increased appetite | **-** | 2 (1.4) | Mirtazapine 2 |
| **Immune system disorders** | **-** | **2 (1.4)** |  |

ADR = adverse drug reaction.

^a^Representing ≥1% of all or preventable ADRs. According to the System Organ Classes according to the Medical Dictionary for Regulatory Activities (MedDRA) [46]. For 11 (1.30%) ADRs, the System Organ Class could not be determined.

^b^Representing ≥1% of all or preventable ADRs. According to the Preferred Terms of the Medical Dictionary for Regulatory Activities (MedDRA) [46].

^c^Chemical substances (5^th^ level) of the Anatomical Therapeutic Chemical (ATC) Classification System [43]. As multiple drugs may be associated with one ADR, the number of drugs may exceed the number of ADRs.
